# Chitosan Schiff-Base Hydrogels—A Critical Perspective Review

**DOI:** 10.3390/gels8120779

**Published:** 2022-11-28

**Authors:** Ioana A. Duceac, Sergiu Coseri

**Affiliations:** P. Poni Institute for Macromolecular Chemistry, 700487 Iasi, Romania

**Keywords:** imine, hydrogel optimization, periodate oxidation, dialdehyde polysaccharide

## Abstract

Chitosan is quite a unique polysaccharide due to the presence of the amine groups naturally occurring in its structure. This feature renders it into a polycation which makes it appealing for preparing polyelectrolyte complexes or imine bonds gels. Therefore, the vast majority of hydrogels prepared using Schiff base chemistry have chitosan as one component. Usually, the counterpart is a low molecular weight aldehyde or a macromolecular periodate-oxidized polysaccharide, i.e., cellulose, pullulan, starch, alginate, hyaluronic acid, etc. Indisputable advantages of hydrogels include their quick gelation, no need for crosslinking agents, and self-healing and injectability properties. This gives grounds for further research, both fundamental in materials science and applicative in various domains. This article is a critical assessment of the most relevant aspects of this topic. It also provides a short review of some of the most interesting research reported in the literature supporting the main observations of this perspective.

## 1. Historical Background

Hugo Schiff, then 30 years old, made the discovery that aromatic aldehydes and primary amines can combine to form imine derivatives in 1864 [1]. A > C=N imine bond has the unusual characteristics of being both reversible, via a quick hydrolytic process, and strong, as predicted for a covalent double bond. Due to these characteristics, Schiff base condensations are thermodynamically regulated, enabling the synthesis of sophisticated and complex structures by a system of trial-and-error in reactions combining multifunctional aldehydes and primary amines. By hydrogenating C–N bonds under benign conditions, back hydrolysis can be avoided. Stable rings and cages of various sizes can be created in this way. Additionally, Schiff base condensations of even more complicated chemical complexes, whose structure is governed by the geometrical preferences of the metal, can be addressed by transition and post-transition metal ions by creating coordinative interactions with imine nitrogen atoms [2]. Multinuclear metal complexes with the shapes of double helices, tetrahedral containers, and, most amazing of all, Borromean rings have been created by Schiff base condensations [3]. Although these molecular creations cannot be compared to the great works of art in painting and sculpture found in the surrounding world, they nonetheless provide the spectator a sense of aesthetic pleasure and respect for the individuals who created them. 

A Schiff’s base is a nitrogen analogue of an aldehyde or ketone in which the carbonyl group (>C=O) has been replaced by an imine or azomethine group. It is also referred to as an imine or an azomethine. The biological actions of Schiff’s bases have also been demonstrated to span a wide range, including antifungal, antibacterial, antimalarial, antiproliferative, anti-inflammatory, antiviral, and antipyretic effects [2]. Various natural, naturally derived, and synthetic substances have imine or azomethine groups. It has been demonstrated that the imine group in the structure of these compounds is essential for their biological actions. Schiff’s bases are significant substances because of the numerous industrial uses they have. By adding Schiff’s bases to polymer films, it is possible to increase poly(methyl methacrylate) resistance to degradation [4], protect polystyrene from photodegradation [5], and photostabilize poly(vinyl chloride) polymers against photodegradation by ultraviolet radiation [6]. A seemingly endless list of records occurs when you search for “Schiff bases” in any chemistry database, demonstrating the significance of such derivatives in chemistry. They serve as reactants in a large number of synthetic organic processes, crucial scaffolds in organometallic chemistry, the structural core of priceless catalysts, and pharmacological presidiums against a variety of various diseases and pathological conditions.

## 2. The Rationale for the Interest in Chitosan Schiff-Base Hydrogels

Due in large part to the novel properties that these compounds meet, early research in the field of reactions with Schiff base production focused exclusively on low molecular weight compounds. Moreover, polymers could not yet be discussed because organic chemistry was still in its infancy. However, when polymers became more prevalent in society and their practical significance in a wide range of sectors came to light, this fundamental reaction in classical organic chemistry took on new significance and produced some of the most stunning outcomes.

It goes without saying that in the modern era, the enormous interest in natural polymers, as substitutes for synthetic polymers, directed research towards new materials, with superior properties, with better mechanical resistance, with characteristics such as self-healing, stretchability, flexibility, self-adhesive, resistant to low temperatures, for areas of use that did not exist a few decades ago, such as human motion monitoring, electronic wearable devices, hardware in computers and other electronics, smart cars, energy storage. A special place among these materials belongs to hydrogels.

### 2.1. Hydrogels

As a type of polymeric biomaterial, hydrogels can be fabricated to have impressively high absorbing degrees, specifically tailored surfaces which are generally highly biocompatible in various biological environments, similar texture and mechanical properties to extracellular matrix (ECM) and soft tissues [7]. Nevertheless, limitations can be pointed out depending on the perspective: low mechanical resistance, difficulties concerning purification and degradation behaviour, injectability and self-healing (occurrence or lack thereof), mass transfer correlated to the swelling and molecule release rates and mechanisms, etc.

In opposition to the rather simplistic classification of hydrogels into physical and chemical, referring to the nature of the crosslinks as non-covalent and covalent, the overwhelming amount of literature and research that exists on this type of material is proof of how many polymers can be used when fabricating a hydrogel and how much the inter-catenary interactions can vary or combine, thus leading to stable, highly hydrophilic networks.

Conductive hydrogels have the potential to self-heal when injured by outside pressures, providing improved levels of safety, dependability, and longevity. Dynamic covalent linkages or non-covalent bonds are frequently included during the creation of a self-healing hydrogel. Schiff base bonds, disulfide bonds, borate ester bonds, and Diels–Alder reactions are examples of dynamic covalent bonds [8]. Hydrogen bonds, metal coordination bonds, host–guest interactions, and hydrophobic interactions are examples of non-covalent bonds [8]. These linkages can be repeatedly broken and repaired, giving the hydrogel the necessary self-healing properties. However, it is typically challenging to achieve high mechanical strength for self-healing conductive hydrogels because there is a natural trade-off between dynamic crosslinking for healing and stable crosslinking for mechanical reinforcement. Researchers frequently choose the methods based on dynamic covalent connections using Schiff base bonds as their first choice from among the several approaches used to manufacture hydrogels.

### 2.2. Schiff-Base Linkage in Hydrogels

**Figure 1:**
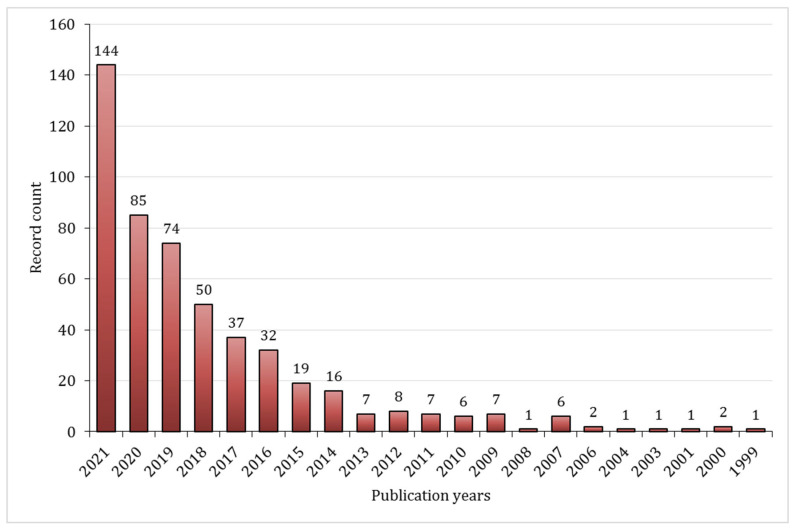
Number of published documents between 1999 and 2021, based on the data from Web of Knowledge, query “*Schiff-base hydrogels*”. Data retrieved 30 September 2022.

The number of articles on the topic has significantly increased in the last decade, as indicated by the statistical data from the web of knowledge illustrated below, Figure 1. Moreover, almost 80% of the articles published on this subject fit into one of two categories: polymer science or materials science.

Most of the interest concerning these materials is focused on biomedical applications for two main reasons: they are injectable and self-healing gels [9]. The former is a highly suitable solution for 3D printing and clinical challenges (such as the surgical intervention required for implanting a medical device for drug delivery or tissue reconstruction) since it is based on an in situ crosslinking reaction, which is undergone quickly, easily and safely. The latter is most promising for cell encapsulation and tissue engineering applications.

### 2.3. Chitosan in Schiff Base Hydrogels

Chitosan is a polysaccharide that has gained increasing attention in the past decades. It is the second most abundant after cellulose and is obtained from marine waste by chitin deacetylation. In contrast to the initial polysaccharide, chitosan is highly reactive due to the presence of the amino groups on its chains, which renders it an attractive biopolymer. This rather unique feature in the class of carbohydrates gives grounds for the intensive use of chitosan for the preparation of various materials for any number of applications [10]. Ionic interactions [11] and polyelectrolyte complexes [12] were successfully used to prepare chitosan-based hydrogels for the controlled release of biologically active agents. Chemical crosslinking was also explored either by grafting or synthesis of semi-interpenetrated networks using low molecular weight compounds or in-situ polymerization [13,14,15,16]. However, self-healing chitosan hydrogels based on Schiff base linkages have attracted a lot of attention. They are easily obtained using chitosan as one partner and an aldehyde bearing compound as another. On this type of material, authors have reported on hydrogels with various types of compositions, which are further discussed.

## 3. Fundamental Research: Focus on Material Science

### 3.1. Injectability and Self-Healing Behavior

One of the most critical parameters of injectable hydrogels is the gelation time. The extreme cases are: either the gelation occurs too slowly, and the gel is delocalized due to the diffusion of the polymers, or the gel precursors crosslink too fast and the gel clogs the needle, never to be injected. Exploring gelation can easily be conducted by means of rheology, particularly time sweep experiments. Moduli evolution in time indicates the behaviour of networks, particularly ones based on dynamic bonds. The gelling point corresponds to the intersection of the curves when the storage modulus G’ becomes bigger than the loss modulus G’’. In addition, injectability can also be explored using flow curves, i.e., viscosity and strain variations with the shear rate. Rheology data is often supported by optical or microscopy images to confirm material stretching, healing or failing. It is noteworthy that water is a must-have for rheology analysis and self-healing behaviour.

The spontaneous regeneration of structure is a biologically-inspired phenomenon that was successfully translated to intelligent polymer materials. The self-healing ability was mostly implemented to hydrogels and constitutes a remarkable progress for areas such as wound dressing, drug delivery systems, sensor, tissue fillers and reconstruction [17]. In developing Schiff-base hydrogels, the self-healing behaviour is readily influenced by any number of factors. These variations can be divided into two categories: factors relating to structure and composition, and external factors, i.e., solution parameters, temperature, freeze-thawing cycles, etc. Perhaps the most significant influence on the self-healing ability of a chitosan-based hydrogel is the amino/aldehyde ratio. An increase in CHO ratio may occur due to a higher concentration in the aldehyde-bearing compound or a more intense oxidation reaction of the chosen polysaccharide. It was observed that, with the increase in aldehyde concentration, the network becomes stiffer and consequently the material exhibits a lower self-healing ability [18,19,20,21,22,23,24]. Moreover, some variables such as polymer concentration and solution pH have also been studied. In a paper by Craciun et al. [23], the hydrogels were tuned in terms of water/solid content; the rheological studies indicated that the recovery properties increased along with the increase in water content. In another article, the authors reported on the influence of the pH and incubation time on the self-healing properties [19]. It was observed that a prolonged incubation may induce a “maturation” effect, similar to a supplementary aldehyde content. The reaction continues as long as the conditions are favorable and there are available moieties. However, this phenomenon has a negative impact on the recovery properties of the material. Interestingly, the pH has a different impact on the hydrogel’s behavior. In the acidic range, the Schiff-base bonds break and the network disintegrates; at alkaline pH, the hydrogel becomes too weak. Nevertheless, at neutral pH the hydrogel can self-heal and fully recover its shape.

### 3.2. Optimizing the Amine/Aldehyde Ratio

Numerous studies reported in the literature explored the influence that the amine/aldehyde ratio has on the properties of the final material. This variation can be performed by modifying the weight ratio of the dialdehyde polysaccharide [25] or by changing the oxidation degree of the polymer [20]. Either way, it has been observed that an increase in CHO groups leads to major modifications in hydrogel behaviour: increase in mechanical resistance, increase in self-healing/recovery time (due to less amine groups available for new imine bonds and, therefore, less crosslinks), increase in storage modulus G’, i.e., mechanical resistance and rigidity. In opposition, some parameters decrease: pore dimension, swelling degree, cell viability, blood coagulation degree, gelation time, and water diffusion coefficient.

### 3.3. Optimizing the Solution Concentration

It has been noticed that the variation in concentration for the polymer solutions has a major impact on hydrogels prepared by this method; a decrease in chitosan concentration has similar impact to an increase in –CHO ratio [23]. Moreover, a lower polymer concentration may lead to better self-healing behavior and injectability.

However, the decrease in consistency and mechanical resistance may impair their use for certain applications, such as joint replacement. It was also found that if the gelling does not occur soon after the polymer is injected, the macromolecules are dispersed in the biological environment and the implantation fails. Therefore, the best self-healing material may not be the most suitable one.

### 3.4. The Niche of Composites and Hybrid Materials in Schiff-Base Hydrogels

Magnetic nanoparticles and ceramics such as hydroxyapatite have been incorporated into injectable hydrogels and the promising results cannot be overlooked. The need for dental and orthopaedic solutions that are clinically effective and financially affordable is still an open door for research. Research has looked into injectable materials with the role of cement tissue fillers, sealants, or even drug delivery systems with magnetic particles embedded into polymeric matrices with Schiff-base crosslinks [26].

Another interesting research direction is the fabrication of “plum pudding” hydrogels. These materials are hybrids that consist of nano- or micro-particles, such as liposomes, nanogels, or polymeric particles, which are physically or chemically entrapped inside the bulk hydrogel network [27,28]. With the ability to independently control and tune both components, one can finely tailor a drug delivery system with controlled/prolonged release that can be easily administered by injection. In our group, for example, the Schiff-base reaction was employed as coating for chitosan-based hydrogel beads, aiming to achieve a controlled release of various anti-inflammatory and antibiotic drugs, such as ibuprofen, bacitracin and neomycin. Differences in the coating polymer led to fine-tuning the porosity and, therefore, a different drug release profile and mechanism [29].

### 3.5. Crosslinking Conditions

Many articles report on the preparation of Schiff base-based hydrogels under simulated physiological conditions, i.e., neutral phosphate buffer saline (PBS) and 37 °C [28,30,31]. Therefore, one might ask: are they absolutely necessary or are there any essential conditions for preparation of these hydrogels? Such hydrogels can be successfully obtained in distilled water at room temperature [19,20,21], as well as in the complex environment that is the biological milieu. Obviously, stability is a major concern that will be further discussed.

However, it must be noted that these are dynamic crosslinks and therefore the hydrogels undergo structural modifications in time [23]. As the material “matures”, more crosslinks are formed and the network becomes denser and stiffer; macroscopically, the gel gets tougher and a change in color may also be noticed.

### 3.6. Crosslinking Alternatives–Variations in the Crosslinking Agent/Polymer Pair

Low molecular weight crosslinking agents ensure fast and easy gelation but raise purification issues due to their toxicity. The most common bifunctional crosslinking agent used to be glutaraldehyde since it readily leads to the formation of hydrogels. However, issues related to high degrees of toxicity have majorly impacted its use, to the point of exclusion from the area of materials for biomedical applications.

Monoaldehydes can also be used to form chitosan-based hydrogels, which involve multiple physical interactions to form stable networks. The topic has been intensively and extensively studied by the group of L. Marin with promising results in various domains, using a large number of aldehyde-bearing compounds: pyridoxal 5-phosphate [23,32], acetaldehyde, citral [33], 2-formylphenylboronic acid [34], salicyladehyde [35], 5-methoxysalicylaldehyde [36], nitrosalicylaldehyde [37], vanilin [38], cinnamaldehyde [39] and others [40].

Although glutaraldehyde has gained a pariah status among crosslinking agents, any unreacted aldehyde groups may lead to cytotoxicity. In order to ensure the safety and biocompatibility of the hydrogels, it is imperative/of paramount importance to either remove these groups by thorough purification and elimination or to compensate the aldehyde excess with another compound [41].

Chitosan can form crosslinked networks without the presence of a low molecular weight crosslinking agent. In this case, there are the so-called zero-length crosslinks between complementary groups pendant on the macromolecular chains of different polymers; the reaction occurs directly at the contact between the two polymers. Aldehyde-bearing high molecular weight compounds used for the preparation of hydrogels with imine bonds are mainly periodate-oxidized polysaccharides, which are easy to oxidize and use, ensure fast gelation, are reliable and eco-friendly.

### 3.7. The Influence of the Oxidized Polysaccharide

**Figure 2:**
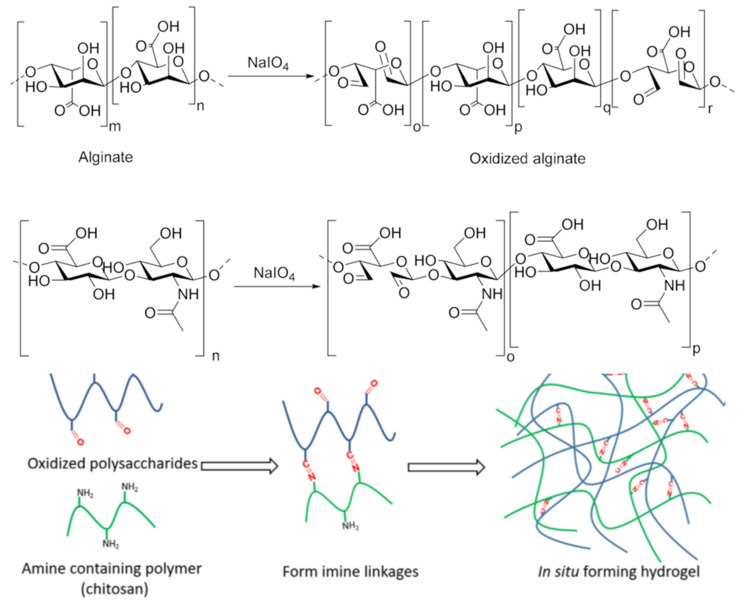
Periodate oxidation of polysaccharides exemplified on two of the most representative examples, i.e., alginate and hyaluronic acid [46], and a typical representation of an in-situ forming hydrogel via Schiff base reaction.

Chitosan is frequently utilized as a starting material in Schiff base formation processes to create cross-linked hydrogels because it has NH_2_ groups. In order to achieve the ultimate goal of linking the NH_2_ bonds of chitosan with aldehyde-type bonds, numerous “*partner*” polysaccharides were thus envisaged and accurately produced. In this regard, it seems particularly advantageous to use sodium periodate, a specific reagent, to oxidize the OH groups contained in the majority of polysaccharides. The unique property of this selective and specific reagent is that it simultaneously oxidizes the secondary OH groups of the glycosidic unit and breaks the bridge between C2 and C3, resulting in a reaction product with two aldehyde groups on the structural unit [10,29,42,43,44,45], Figure 2.

Numerous variables must be taken into consideration when analyzing the polysaccharide formation reaction, including the ratio of periodate to polysaccharides (in general, periodate is used in large excesses due to the low reactivity of the secondary OH groups), temperature, and the type of polysaccharide. The degree of oxidation was higher in oxidized hyaluronic acid than in oxidized alginate, and it was observed that the molecular weights of all oxidized polysaccharides decreased with oxidation. Notably, oxidized polysaccharides with an oxidation level exceeding 25% were found to be cytotoxic, according to studies [46].

### 3.8. Fine-Tuning Chitosan: Argument for Its Chemical Modification

The main difficulty in working with chitosan is its solubility in water at neutral pH. This is in addition to possible unwanted interactions that may appear as a consequence of the high reactivity of amino groups in contrast with the hydroxyls. Numerous strategies have been sought and tried with favourable results. The major advantage is the ease of processability—no acid required for water-based solutions. Among the possible derivatives that have been studied in the composition of Schiff base hydrogels are: N-succinyl-chitosan [22,30,47,48], quaternized chitosan [31], N,O-carboxymethyl chitosan [18,28,49], acrylamide-modified chitosan [19], and glycol-chitosan [50]. Interestingly, depending on the derivative, the network evolves differently in time. It was observed that N-substituted chitosan can lead to an increase in crosslinking density over time, while N,O-substituted chitosan may exhibit the opposite behaviour [51].

### 3.9. The Amine Bearing Component: Alternatives to Chitosan

Hydrogel networks with imine crosslinks have also been prepared without chitosan. For example, gelatin is a suitable candidate for interactions with oxidized pullulan [52]. Such hydrogels were extensively studied for tissue engineering applications and exhibited adequate mechanical strength and biocompatibility. The material was successfully seeded with chondrocytes and enabled cell proliferation and production of the cartilage-specific matrix.

Another chitosan alternative that can be considered is a low molecular weight compound, i.e., ethylenediamine. Microgels based on dialdehyde dextran were prepared by the water-in-oil inverse microemulsion method. The authors used a –NH_2_/–CHO molar ratio of 1/2 [53].

Recently, in our group, a new type of Schiff-base hydrogel was obtained based on dialdehyde pullulan [42]. Dopamine was selected as a low molecular weight compound bearing an amine group that attached to the aldehyde moieties on the polysaccharide backbone and enabled the formation of a stable network.

### 3.10. The Issue: The pH Stability and Degradation Behaviour of Schiff-Base Hydrogels

Several articles have tapped into this issue and reported on the behaviour of Schiff base hydrogels under pH variations. The results indicated a loss of integrity around pH = 5, which is unanimously accepted. The mass loss of chitosan-based materials at pH around 5 is in accordance with the well-known dissolution of this polysaccharide due to the protonation of amino groups [54]. In addition, an improvement was noted regarding chitosan beads’ stability due to crosslinking with periodate-oxidized cellulose or pullulan [29,54]. However, gel formation at acidic pH was observed; imine bonds are not stable in such a medium, so another mechanism was searched to explain network formation. Considering the high concentration of H^+^ in the environment, scientists hypothesized that a large number of hydrogen bonds could form [21]. Consequently, that will allow the polymer to be stabilized into a 3D network and the material would regain its integrity and solid-like behaviour.

## 4. Applicative Research: Focus on Biomedical Technologies

As compared to other dynamic bonds encountered when preparing hydrogels, the Schiff-base reaction occurs fast, easily and under mild conditions without metal catalysts, temperature or other reaction conditions. The imine bond enables pH-responsiveness and reversibility rendering in hydrogels with attractive properties for numerous applications: drug delivery, wound dressing, tissue reconstruction, bioprinting, tissue adhesive, biosensors, etc. [9].

Hydrogels are one of the most important types of vehicles for drug delivery. Chemotherapy can be successfully undergone by local treatment using hydrogels. However, a whole new perspective opened with injectable hydrogels. With a minimally invasive procedure, soft materials can be implanted and may ensure a prolonged release profile of the loaded drug [27,29].

A major advantage of these hydrogels in the area of tissue engineering and 3D printing is the possibility to include viable cells prior to injection or printing. Numerous studies reported on the successful inclusion of different cell lines into chitosan-based hydrogels stabilized by Schiff-base bonds under static conditions or during extrusion [55,56].

Wound healing is another area of great interest and concern. Materials based on imine bonds were prepared for wound dressing applications and were proved to exhibit more than one feature of interest for this application. For example, hydrogels based on oxidized polysaccharides are proved to have an excellent hemostatic effect [42]. Moreover, the presence of bioactive molecules can accelerate the tissue repair and the wound closure [57].

## 5. Final Remarks and Future Perspectives

Numerous investigations on hydrogels reveal that they are a prime choice for use in biomedical applications. In terms of structural qualities, mechanical properties, and biological properties, natural extracellular matrices can be accurately replicated. The hydrogel’s self-healing abilities can increase the useful life of products, conserve resources, and safeguard the environment. Self-healing hydrogels, which outperform conventional hydrogels in terms of durability and service life, are frequently employed in 3D printing, wound dressings, drug delivery, and cell culture. The hydrogel’s biological features should be taken into account in addition to its capacity for self-healing. This implies that the raw materials chosen ought to be biocompatible and biodegradable. The majority of polysaccharides, such as cellulose, alginic acid, hyaluronic acid, and chitosan, are derived from natural plants or animals and are notable for the abundance of hydroxyl, carboxyl, and amino groups in their structural chains. The added capabilities enable customized performance, which is frequently prompted by stimuli, including temperature, UV and NIR light, pH, enzymes, and reactive oxygen species. However, obstacles still stand in the way of using hydrogels made of polysaccharides in situ for biological purposes. One concern is possible toxicity. Newly introduced motifs and their breakdown products may raise unanticipated toxicity problems for hydrogels made from modified polysaccharides. Furthermore, this particular hydrogel has not yet fully realized or has room to further develop a number of desirable features. For in-situ-forming hydrogels, appealing properties include quick, regulated gelation and quick self-healing. These groups are crucial for the development of reversible covalent and non-covalent bonds. Self-healing is typically accomplished in the creation of self-healing hydrogels by altering the bonding mechanism of the polymers and adding sacrificial linkages. Natural polysaccharides benefit from being abundant sources, having good biocompatibility, and being biodegradable, all of which support the use of hydrogels based on polysaccharides in biomedicine. We can draw some inspiration from the creation of intelligent hydrogels to include groups or compounds in the design of self-healing hydrogels that can react to light, heat, electricity, and other environmental stimuli. Once the hydrogel has the ability to respond to stimuli, modifying the environment can have an impact on the hydrogel’s mechanical and self-healing characteristics. Despite the recent advancements in self-healing hydrogels, there are still certain difficulties to be resolved. In general, a hydrogel’s mechanical qualities will decline when its self-healing ability is increased. Therefore, designing hydrogels that can simultaneously increase their self-healing and mechanical properties should be taken into account. Since the conducting components in the conductive hydrogel are often nonbiodegradable and there is a safety hazard problem in long-term use, the application of self-healing hydrogel in 3D printing and wearable electronic equipment also requires more test evaluation. Self-healing hydrogels can keep biological scaffolds intact, but their strength is typically not very high. An urgent topic in the field of tissue engineering is the creation of self-healing hydrogels with high strength. Chitosan appears to have taken on the primary role of the component supplying NH_2_ groups, being the first choice for coupling with polysaccharide components carrying aldehyde groups. Research on hydrogels based on imine bonds is reaching its pinnacle. However, we think that new materials with unexpected properties can be made using chemical functionalization techniques for the selective and directed twisting of amino groups in other kinds of polysaccharides, followed by the reaction with aldehyde groups present either in low molecular weight compounds or polymers. In conclusion, the use of hydrogel in biomedical materials will be able to grow to clinical applications due to the demand for advanced biomedical materials and the ongoing development of hydrogel research.
